# Revision Endoscopic Gastroplasty: An Overview and Review of Literature

**DOI:** 10.7759/cureus.42099

**Published:** 2023-07-18

**Authors:** Basil N Nduma, Kelly A Mofor, Jason Tatang, Loica Amougou, Stephen Nkeonye, Princess Chineme, Chukwuyem Ekhator, Solomon Ambe

**Affiliations:** 1 Internal Medicine, Merit Health Wesley, Hattiesburg, USA; 2 Gastroenterology, Paul L. Foster School of Medicine, El Paso, USA; 3 Gastroenterology, Sam Houston State University, Huntsville, USA; 4 Gastroenterology, School of Natural Sciences and Mathematics, University of Texas at Dallas, Richardson, USA; 5 Oncology, University of Texas Rio Grande Valley, Houston, USA; 6 Gastroenterology, University of Texas at San Antonio, San Antonio, USA; 7 Neuro-Oncology, New York Institute of Technology College of Osteopathic Medicine, Old Westbury, USA; 8 Neurology, Baylor Scott & White Health, Dallas, USA

**Keywords:** bmi (body mass index) loss, suboptimal weight loss, revision endoscopic gastroplasty (r-esg), roux-en-y gastric bypass (rygb), weight recidivism, safety, efficacy, revision endoscopic gastroplasty

## Abstract

The main aim of this paper was to examine the efficacy and safety of revision endoscopic gastroplasty and some of the adverse events likely to arise from the procedure, as well as the implications for future scholarly research. The study is a systematic review in which the PRISMA protocol was used to govern the article's inclusion and exclusion criteria. The selected studies include those on revising endoscopic gastroplasty's effectiveness and safety. The studies were selected based on multiple parameters. The outcome included weight recidivism, excessive BMI loss, and absolute, total, or percentage weight loss. The outcome of this review confirmed that revision endoscopic gastroplasty is effective and safe. Mainly, revision endoscopic gastroplasty (R-EG) was found to counter-weight recidivism, especially short-term and mid-term. However, there is a need for additional scholarly investigations that would last several years to decades to inform the long-term efficacy of R-EG with precision.

## Introduction and background

Currently, the incidence and prevalence of obesity have reached pandemic proportions. To mitigate the condition, several techniques have been embraced. To date, bariatric surgery has dominated these strategies due to its associated effectiveness [1]. Among bariatric surgical procedures, the most frequently performed procedure worldwide is laparoscopic sleeve gastrectomy (LSG). The dominance of LSG accrues from the position that it proves technically easier to implement and comes with weight loss outcomes similar to other procedures, such as Roux-en-Y gastric bypass (RYGB) [2]. In LSG, there is resection of the stomach's greater curvature, and the aim is to ensure volume reduction by close to 80%. At one-year post-procedure, LSG has been linked to excess weight loss (EWL), approximately from 48% to 72%. In addition, obesity-related comorbidity improvements were also documented, which included hypertension, dyslipidemia, diabetes, and obstructive sleep apnea, in which durable mid-term outcomes were reported [3]. However, weight recidivism tends to result following LSG implementation in some subsets of patients. This situation is most vivid in sleeve dilation contexts. At seven or more years of follow-up, insufficient weight loss (less than 50% EWL) or weight gain has been avowed to arise in about 14% to 37% of patients [4]. In response, revision rates were estimated to be about 13% at about the same time. The implication is that for weight recidivism, LSG revisions have mostly assumed the repeat surgical sleeve approach, duodenal switch, and conversion to RYGB [5-9]. Although these revision interventions are currently widely being implemented, there is still a paucity of data in determining optimal revision procedures. In particular, the respective revision procedures are associated with significant morbidity rates, with the case of surgical revision of LSG coming with overall rates of adverse events stretching between 5% and 20%, which is significantly greater compared to risks associated with the initial surgical interventions [9]. Obesity is associated with chronic relapses following LSG, a trend that comes at a time when the popularity of LSG is growing and seeing an increase in the number of younger patients being offered the procedure. This trend has created a huge demand for safer revision options. The increase in the demand is informed by the need to ensure weight loss induction and obesity-related comorbidity remission following a decreased efficacy of primary LSG [5,9].

To mitigate the obesity pandemic's widening management gap, endoscopic, metabolic, and bariatric therapies have evolved. These procedures are applied to manage weight recidivism following RYGB implementation. On the one hand, endoscopic gastric suturing (EGS) may not necessarily induce significant weight loss compared to its traditional surgical counterpart regarding obesity's primary treatment. On the other hand, using the endoscopic technique for dilated LSG volume restriction for managing weight regain or suboptimal weight loss remains an appealing option [10-19]. Indeed, R-EG (revision endoscopic gastroplasty) has been associated with numerous benefits compared to traditional surgical revision techniques. Some specific benefits linked to R-EG include the organ-sparing nature that provides room for additional revision surgery in the future, technical ease, and improved safety [19]. These observations suggest that when the LSG revision landscape remains poorly understood in most cases [6], R-EG emerges as a promising direction that might serve a crucial role [7-8].

Aimed at contributing to the literature and responding to the perceived knowledge gaps, this paper's central purpose was to examine the efficacy and safety of revisional endoscopic gastroplasty from a systematic review perspective, drawing insights from selected previous scholarly research studies that had focused on this subject. The motivation of the review paper was to predict optimal revision procedures likely to ensure better patient outcomes, proceeding further to discuss the emerging implications for the future of healthcare or clinical environment activities revolving around obesity management, especially weight recidivism management.

## Review

Materials and methods

A systematic review approach was adopted. The eligibility criteria for inclusion into the study were clearly outlined. Particularly, the articles selected for use in the review include peer-reviewed documents centered on the utilization of revision endoscopic gastroplasty. The articles for inclusion were also expected to have been published in English and documented as full-text materials within the past decade. In scenarios involving articles appearing in different search engines or databases, a consensus was achieved by the reviewers. Hence the eligibility criterion at this stage was coined to hold that articles appearing on different websites would be ascertained and redundancy eliminated, ensuring further that if the authors had focused on the same subject and with similar subjects, only articles constituting the most subjects would be considered for use in the review article.

The selected studies' research designs were also considered to determine their inclusion or exclusion as required. Some of the included studies were those conducted in the form of randomized clinical trials, cohort studies, case series, prospective non-randomized trials, case-control studies, and case reports. The eventuality was that articles appearing in forms such as guidelines, paper comments, letters, brief reports, and protocol studies were considered as not satisfying the inclusion criteria relative to the research design consideration aspect, hence their exclusion from the current study. Apart from the research designs, the research participants in the respective chosen studies were also considered in the inclusion criteria. Here, the selected articles centered on individuals of all ages in which revision endoscopic gastroplasty was implemented. The research duration was also a factor considered during article selection. In this case, both long-term and short-term intervention studies were considered; hence the inclusion of those whose follow-ups were for a few months and those whose follow-ups to determine revision endoscopic gastroplasty's efficacy and safety were for a year or more. The decision to include both short-term and long-term interventions and follow-up studies was to ensure better insight into whether revision endoscopic gastroplasty could be recommended for patients with different magnitudes of weight recidivism.

Still, the methodology considered the intervention type and the predictive and outcome variables. Here, the key intervention focused on the predictive variable included the implementation of revision endoscopic gastroplasty. On the other hand, the articles with outcome variables of effectiveness or safety of the intervention mentioned above were included as part of the inclusion criteria. The specific aspect that would inform intervention effectiveness involved the capacity to mitigate weight recidivism or ensure excessive BMI loss, absolute weight loss, total weight loss in percentage, and excess weight loss in percentage. The implication is that a study reporting one or more of such outcome measures was eligible for inclusion. Beyond these primary outcomes, during the selection of articles for inclusion in the systematic review, there was further focus on specific secondary outcomes that the researchers reported. These included articles were those whose authors strived to give insight into the correlation between revision endoscopic gastroplasty and the occurrence of adverse events in the intervention or experimental groups. The objective of considering this factor during article searches in databases was to ensure the ability to predict the procedure's safety, hence recommendations for or against large-scale adoption of the intervention in clinical contexts. Still, with secondary outcome reporting in mind concerning factors informing article selection, this review included scholarly studies that gave insight into the patients' risk factors that could attract adverse events following revision endoscopic gastroplasty implementation.

**Figure 1:**
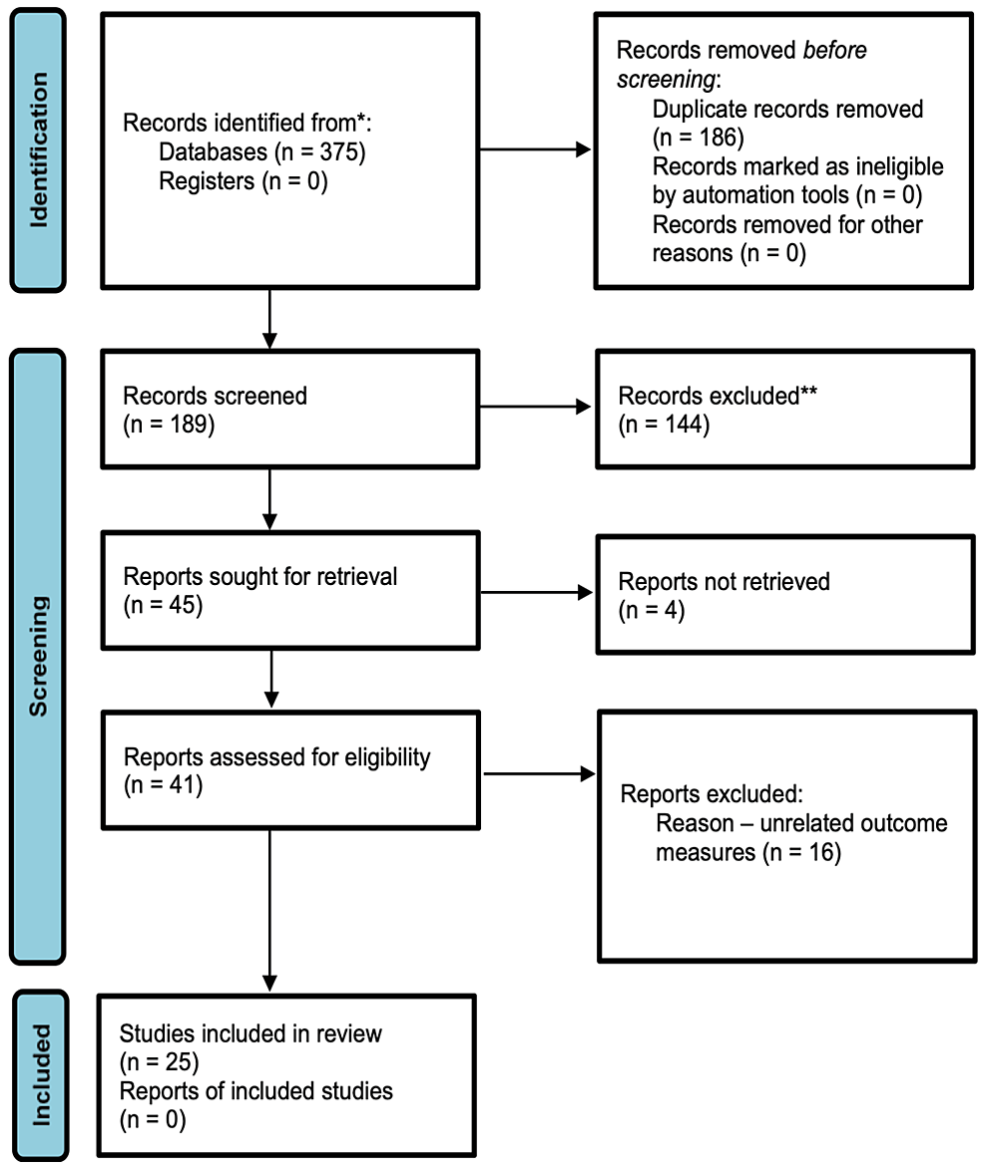
The PRIMA flowchart for systematic reviews

Morbidity alteration was another attribute that was considered during the article search process. For instance, the articles were selected and analyzed based on the outcomes concerning the ability of the selected procedure to yield improvements in patient quality of life and the ability to yield reductions in obesity-related comorbidities such as hypertension and obstructive sleep apnea. Once the above considerations were completed, the following databases were utilized for searching and accessing articles: MEDLINE, EMBASE, and Clinicaltrials.gov. The period of investigation was in March 2023. Initially, titles and accompanying abstracts were screened independently, utilizing keywords such as revision endoscopic gastroplasty, efficacy, safety, weight recidivism, RYGB, R-EG, suboptimal weight loss, and BMI loss. Full articles were assessed after title and abstract evaluation to see if they satisfied the inclusion criteria. In case of differences in the reviewers' opinions concerning the eligibility of some articles, there was an eventual consensus among authors, as mentioned earlier. Notably, the PRISMA frame for systematic reviews was used to guide the selection of articles. The process is outlined in Figure 1 below, and it involves the search and identification of articles, screening, determination of eligibility, and inclusion or otherwise. The following figure summarizes the PRISMA framework used in this article. 

Result

Table 1 below outline the articles included in this study.

**Table 1:** Table outlining included studies.

Reference	Study Aim	Results	Clinical Implications
Gallo et al (2016) [9]	To analyze the outcomes after restorative obesity surgery, the endoluminal (ROSE) procedure	At one year, a sustained weight loss was reported for all patients following reoperation or resleeve.	Revision endoscopic plication depicted promising outcomes, expanding knowledge on the future of weight gain or regain management via reoperation.
Vargas et al. (2018) [10]	To examine long-term efficacy and the efficacy of resleeve following weight regain in patients following bariatric surgery.	Reoperation, when applied alongside multidisciplinary intervention, was found to be effective, reproducible, and safe.	Weight recidivism could be managed effectively via reoperation, hence the need to utilize resleeve early.
Sharaiha et al. (2015) [11]	To determine the effectiveness of revisional ESG after reoperation in weight recidivism cases.	Findings held that after sleeve gastrectomy, successful sleeve plication results, with specific outcomes demonstrating a value of 9 kg as the induced weight loss.	In the future, larger cohorts will be worth examining, and also longer durations of follow-up will be used to gain more specific insights into revisional ESG’s perceived efficacy.
Eid (2017) [12]	In patient cases with an enlarged gastric sleeve, revisional ESG was implemented to establish the impact on weight loss management.	Findings revealed an increase from 6.7 percent to 17.2 percent as the increase in total body weight loss at one year.	Post-procedurally, revisional ESG can be seen to yield promising results relative to the outcome parameter of weight loss, hence the need for further application in the future, particularly with eligible patient groups.
Maselli et al. (2021) [13]	The multicenter study aimed to establish the impact of revisional ESG on weight loss in 82 patients.	The results demonstrated that for individuals experiencing weight recidivism following LSG, the implementation of revisional ESG would lead to over 10 percent total body weight loss in 72.5 percent of patients at six months, as well as over 10 percent total body weight loss in 81 percent of the research subjects at one-year follow-up.	In the short-term, revisional ESG proves an effective and safe weight loss or weight recidivism management approach.
Hourneaux De Moura & Thompson (2019) [14]	To determine the effectiveness and reliability of revisional ESG after RYGB.	The efficacy of revisional ESG was ascertained and associated with the ability to treat weight recidivism arising from dilated pouch outlets.	The results were, however, short- and medium-term, pointing to the need for future studies to ascertain longer-term effects if any.
Bulajic et al. (2021) [15]	To find out the feasibility, selection, and indication of revisional ESG in weight regain management after primary bariatric surgery.	Results demonstrated that weight regain after primary bariatric surgical intervention arises from several factors and, although revisional ESG may be promising, the extent to which a personalized approach could be embraced to satisfy each individual patient’s needs remains unclear.	In the future, it will be necessary to examine how a personalized approach could be optimized relative to revisional ESG implementation.
Callahan et al. (2020) [16]	To determine the ability for weight loss maintenance up to five years postoperative in patients exposed to reoperation.	Findings pointed to a more significant percentage of EBWL and also a greater percentage reduction in the stoma diameter.	Five years after revision, weight loss maintenance was ascertained and there was little impact on medical comorbidities, hence promising outcomes in terms of possible achievement of superior weight loss.
Patel et al. (2017) [17]	The central purpose of the study involved comparing weight loss results in patients undergoing reoperation.	Findings indicated significant comorbid condition resolution, with no major complications arising.	Revisional endoscopic gastrectomy was found to be associated with meaningful resolution of comorbidities and weight loss among patients who have undergone RYGB but still experience weight regain.
Galvão et al. (2019) [18]	To establish the effectiveness of revisional ESG eight months post-surgery.	Reoperation alongside multidisciplinary team interventions were associated with superior results regarding patient recovery.	In situations, where patients fail to meet the bariatric surgery procedure, the study indicated that the easiest and safest option for achieving weight loss entails a second endoscopic gastroplasty.
Kumar and Thompson (2016) [19]	To find out the long-term weight trend after implementing transoral outlet reduction and also predict factors necessitating resleeve.	Transoral outlet reduction was avowed to mitigate weight regain in terms of effectiveness and safety.	Transoral outlet reduction was observed to be an ideal approach worth using alongside multidisciplinary interventions to mitigate weight regain post-RYGB.
Abboud et al. (2022) [20]	The study aimed to uncover short- and mid-term weight loss outcomes in relation to revisional ESG implementation.	In the findings, it was noted that during revisional ESG implementation, optimal patient outcomes tend to accrue from the complementation with a multidisciplinary approach.	In the future, healthcare teams will be worth educating on the correlation between multidisciplinary collaboration and revisional ESG effectiveness.
Lau & Marks (2020) [21]	The study aimed to discern the efficacy of R-ESG in patients pooled from nine different institutions, with the follow-up period being 12 months in which the population at follow-up stood at 47.6% of the initial 82 patients.	In the results, the authors established that 76.9% of the follow-up group exhibited excess weight loss of more than 25%, concluding a state of clinical success relative to the guidelines by the American Society for Gastrointestinal Endoscopy.	The authors in the study held that with the R-ESG procedure being at its infant stage, the presence of a smaller patient population was not unexpected. It was highlighted further that whereas the beneficial effect of fewer adverse events was the key indicator of promising short-term outcomes with which R-ESG could be associated, the long-term outcomes of the procedure are yet to be elucidated, pointing to the need for further investigation around the subject.
Heylen et al. (2011) [22]	The need to understand the efficiency and safety of the OTSC®-clip application in relation to the quest towards the reduction of pouch-outlet in selected cases was the chief motivation of the study, informed by the need to discern how if any, permanent weight control could be achieved.	In the findings, the efficiency and safety of the OTSC®-clip application were demonstrated.	The treatment of weight gain through the OTSC®-clip for revisional endoscopy can be seen to be an effective and reliable approach following gastric bypass, thus ensuring weight gain treatment in relation to a dilated pouch-outlet.
Razzak et al. (2022) [23]	This case presentation entailed a woman aged 60, diagnosed with obesity class III. She also had a history of abdominal surgeries and multiple comorbidities, with weight regain standing at 22 kg.	With revisional endoscopic sleeve gastroplasty performed, having ascertained poor candidacy for surgery, findings suggested evidence of successful antral reduction.	In the selected investigation, no adverse event was reported, suggesting the safety of R-ESG. However, the investigation being a single-case study implied that outcome validity could not be ascertained due to the limitation of the sample size, as well as the highly unique state of the candidate’s demographic features.
Jirapinyo et al. (2019) [24]	This study was also a single case presentation entailing the implementation of R-ESG to understand the procedure’s effectiveness in reversing weight regain following sleeve gastrectomy.	Results demonstrated that R-ESG is an effective and safe endoscopic technique through which weight regain could be treated.	Despite the promising nature of the findings, it can be seen that insights into the long-term outcomes of R-ESG could not be ascertained, if any. Additionally, there was a lack of clarity regarding the criteria for selecting the patient, an area worth improving upon via future research studies.
Dolan et al. (2021) [25]	The study sought to offer a comparison of weight loss profiles and rates of serious adverse events pitting revisional techniques, with endoscopy also on the focus over a five-year period.	In the findings, it was stated that in the endoscopy group, the rate of adverse events was lower than in the case of the surgical group, standing at 6.5% for the case of the R-ESG group and 29.0%in the surgical group. Also, the rate of serious or severe adverse events stood at 0.0% in the R-ESG group but, in the surgical group, the rate stood at 19.4%. Regarding weight loss, however, no significant difference was reported.	The selected study demonstrated that given gastro-jejunal anastomosis, the rate of total and severe adverse events following the implementation of R-ESG tends to be significantly lower, hence the need to embrace the technique in relevant candidates. However, whether these results would hold in a similar cohort size but with varying severities of disease remained unknown.

One reviewed study examined the degree of success of the endoscopic revision of gastric bypass to discern its effectiveness. This study examined 27 patients between May 2008 and November 2013 [9]. This study showed achievement of the intended stoma and pouch diameter reduction at three months but the eventual tending toward the preoperative diameter at 12 months, leading to short-term weight loss; hence the efficacy of the revisional procedure was documented, but its effectiveness in the medium- and long-term was affirmed to face the risk of anatomical failure. In another large multicenter and international study, the efficacy of revisional procedures was examined [10], pitting 130 patients between January 2013 and November 2016. The results demonstrated key adverse events, including pain and nausea, in 18% and 14% of the patients, respectively, ascertaining the effectiveness, reproducibility, and safety of the revisional approach, especially when utilized as part of a multidisciplinary intervention. In addition, another study that focused on 150 patients with transoral gastric reduction after gastric bypass probing for effectiveness and safety yielded 2.6% excess weight loss at one year, 20.0% at two years, and 19.2% at three years [19]. 

In a case report, a 49-year-old lady was exposed to a revisional procedure following weight regain, with subsequent weight loss of 9kg confirming the efficacy of the revision of R-EG [11]. In a related observation, the focus was on a case series involving five patients with 12 months of follow-up, aimed at discerning the efficacy of employing the endoluminal approach with an endoscopic suturing device for re-sleeve via plication. This study established that the procedure led to a 33% mean excess weight loss, with values of 6.7% to 17.2% obtained for the percent total weight loss [12], further confirming the effectiveness of the R-EG. In a nine-center study involving 82 consecutive adults undergoing R-EG, with the period of investigation ranging from March 2014 to November 2019 and the follow-up period being 12 months, the study demonstrated more than 10% total body weight loss in 81% of the patients at 12 months and 72.5% at six months, with over 15% total body weight loss realized in 52.5% of the patients at 12 months and in 43.5% of them at six months [13]. Only one moderate adverse event was reported: a narrowed gastroesophageal junction. This study further confirmed the effectiveness and safety of R-EG. In a case-series analysis involving endoluminal revisions with the reduction of the gastrojejunal anastomosis and the gastric pouch size, populations such as those experiencing weight regain after Roux-en-Y gastric bypass are likely to benefit optimally because the revisional procedure remains less invasive and effective [14]. To shed additional light on this subject, the feasibility of various techniques for weight regain management was investigated [15], with the results demonstrating that after primary bariatric surgery is performed, weight regain remains multifactorial, but revisional strategies pose promising levels of efficacy, feasibility, and safety.

A five-year follow-up was also used to examine endoscopic gastrojejunostomy revision involving 70 patients [16]. In the findings, the percent of excess body weight loss at five, four, three, two, and one year was 23.8%, 21.6%, 14.9%, 19.8%, and 20.6%, respectively. At six months, the value stood at 18.2%, concluding that weight loss is sustained through the revisional procedure for up to five months. A similar investigation focusing on 47 patients was conducted between June 2012 and September 2015 to determine the effectiveness of postoperative revisional interventions for weight loss [17]. In the study, all cases depicted technical success in which stoma reduction was 76.8% and 84.2% in the purse-string suture group and the interrupted suture group, with only 16 comorbid conditions occurring and resolved, hence no significant complications. In a single case of a woman aged 52, following weight gain in five months following endoscopic gastroplasty, the greater curvature was endoscopically resutured eight months after the initial intervention [18]. Following the revisional procedure, there was a total body weight loss of 16%. With no significant adverse event in this patient, it was inferred that the revisional strategy comes with a promising safety profile and desirable procedure results. Lastly, different endoscopic revision options have been examined in different environments in the U.S. after bariatric surgeries involving SG and RYGB [20], with specific findings demonstrating that after SG, endoscopic sleeve plication tends to induce a weight loss as high as 9 kg at three months, and the ability to yield sustained total body weight loss ranging from 6.7% to 17.2%, hence a favorable safety profile due to no serious adverse events and the procedure being a minimally invasive alternative option for managing weight recurrence after bariatric surgery. 

To further understand the ability of R-ESG to bring about adequate excess weight loss in one year while ensuring fewer adverse events, a study that focused on patients pooled from nine different institutions and motivated by the need to understand how durable the revisional procedure could be, was done [21]. This study documented that the key beneficial effect of R-ESG entails marked reductions in the risk of operative adverse events. The authors did not report significant adverse events during the selected one-year study follow-up. Also, R-ESG was avowed to be unique from other procedures such as duodenal switch, RYGB, and LSG revision because it comes without anastomotic lines that yield a risk of leaks. Hence, it was concluded that the R-ESG procedure exhibits a strong potential to benefit patients needing revision following weight recidivism. In particular, R-ESG evolved as a promising alternative for patients at high risk for surgical adverse events. Despite this promising state of the revisional approach in this study, it should be noted that in the pooled population, 92.7% of the patients were female, an outcome that could be deemed to have likely impacted outcome validity, hence the need for future studies to focus on a more diverse population to discern whether or not similar outcomes could be obtained. Additionally, the extent to which the results were likely to give insight into the long-term durability of the R-ESG procedure remained a dilemma, an area worth exploring further in future studies.

In a study using the OTSC®-clip application to determine the efficacy of the revision endoscopy, the motivation was to inform healthcare professionals on how proper maintenance of the restrictive component of the Fobi pouch gastric bypass would contribute to the realization of longer-term weight control [22]. There were 94 patients in the study. These patients had unintended weight gain after gastric bypass. The study sought to understand how efficient and safe the approach of OTSC®-clip plication revision endoscopy could be and to reveal its promising role in treating weight gain following a dilated pouch outlet. The study observed the best clinical outcomes following gastro-jejunostomy narrowing, particularly by placing two clips at opposite sites. Indeed, the promising results were attributed to the affirmation that the procedure reduced the outlet by over 80%. In the initial three months of follow-up following the application of the OTSC®-clip, a value of 29.7 was documented as the mean BMI of the study participants. At about 12 months, which marked the second follow-up after applying the OTSC®-clip, 27.4 was recorded as the mean BMI. It was concluded from the study that revision endoscopy with an application of the OTSC®-clip following gastric bypass tends to come with a promising state of effectiveness and reliability when used in treating weight gain after a dilated pouch-outlet in the short-term and mid-term. There is a need for further studies to confirm its efficacy and safety in the long term. In a case report, a patient not using any prescriptions or over-the-counter products underwent the R-ESG [23]. No procedural adverse events were reported during the follow-up period, including no pain post-procedure. At two months of follow-up, the total body weight loss stood at 6.5%, with a normal tubular stomach also observed. At this point, the safety of R-ESG was inferred.

In another case report, a female patient aged 58 with a history of cholecystectomy and LSG presented with a problem of weight regain following LSG. At the pre-LSG stage, the patient weighed 301 pounds, with 165 pounds recorded as the postsurgical nadir weight. At the time of the case presentation, the patient weighed 201 pounds, implying that the weight regain was as high as 26.5% relative to maximal weight loss. The patient's new BMI was 34.4. Three months after R-ESG, there was a decrease in weight to 185 pounds from the initial 201 pounds. Indeed, the weight change represented a value of 20.5% for excess weight loss, as well as a value of 8.0% for total weight loss. The resulting inference was that R-ESG could be applied successfully as a reliable endoscopic treatment, especially in patients diagnosed with weight regain following LSG. Additional observations in this study held that at a time when there is a rising prevalence of LSG, more and more cases of this nature would be encountered by gastroenterologists. In these cases, R-ESG could be an effective and safe treatment for weight regain in this population. However, the selection criteria for the two case reports were unclear. Furthermore, the outcome generalizability of case reports is not appropriate due to the small sample size of one. Another limitation of the case mentioned above reports was that the studies could not give critical insight into the long-term beneficial effects of effectiveness and safety with which the R-ESG procedure might be associated, hence the need for more studies with longer follow-up periods. 

In another study, the relationship between R-ESG and the occurrence of adverse events, including severe adverse events, was evaluated in 31 candidates at two tertiary referral centers in a retrospective matched cohort study of patients who experienced weight regain after RYGB. Their weight gain was explained to be caused by dilated or incompetent gastrojejunal anastomosis [25]. In this study, the specific objective was to find differences in the total and severe adverse events rate between patients undergoing R-ESG (n=31) and those undergoing surgical intervention (n=31). The study found that in the R-ESG group, the overall rate of adverse events was lower than in the surgical group, standing at 6.5% and 29.0%, respectively. Some specific forms of serious adverse events documented in medical literature in the R-ESG group include gastrointestinal bleeding and gastrojejunal anastomosis.

On the other hand, some specific forms of serious adverse events documented in medical literature in the surgical group include high-grade small-bowel obstruction, incarcerated incisional hernia, gastrointestinal bleeding, ulcers, and gastrojejunal stenosis requiring balloon dilation [25]. In this study, When it came to the subject of severity, which sought to discern the safety of the R-ESG procedure, the study revealed a value of 0.0% as total adverse events. On the other hand, the investigation revealed 19.4% as the value of serious adverse events in the surgical group. Some serious adverse events that were noted included a deep abdominal wall abscess, incarcerated incisional hernia, and postoperative leak. It was then inferred that R-ESG comes with safety profile improvement, especially because of the presence of fewer total and serious adverse events in the R-ESG group compared to the surgical group [25].

Discussion 

For patients seeking to lose weight, the options tend to be confusing because of the existence of numerous approaches that have evolved in the past decade. In most cases, patients have sought to undergo minimally invasive techniques to achieve weight loss, but many of these procedures have eventually progressed to laparoscopic bariatric surgery. The literature holds that reoperation remains a possible option due to weight recidivism, but challenges are also persistent because of issues such as unidentified hardware and even unexpected extra-gastric adhesions. Regarding R-EG, the safety and effectiveness of the procedure have been documented, but some adversities tend to be linked to adhesions, which have either arisen from the prior insertion and subsequent removal of laparoscopic gastric bands or previous EG attempts [6]. However, adhesiolysis remains uncomplicated in reoperation, implying that it is less likely to yield marked challenges to revision surgery. The presence of undetected cinches or clips during LSG forms one of the critical problems because of the associated increase in the likelihood of serious complications, especially if staple misfiring leads to leakages in staple lines, should they go unidentified. Furthermore, it can be observed that a thicker stomach wall could result from intra-gastric balloons [11,14]. 

Emerging as one of the novel bariatric procedures, R-EG has become increasingly popular, a trend attributed to reasonable early loss of weight with which the procedure is associated. The literature points to good short- to medium-term weight loss outcomes and ESG's reversibility as required. However, this review paper projects that the uptake of R-EG as a key bariatric approach will be determined by potential long-term weight loss and safety outcomes, a trend yet to be documented widely, especially in investigations focusing on longer-term follow-up periods extended to several years. There is also a need for future studies to embrace endoscopic evaluations in a quest to assess the anatomical distortion and pathology of the stomach, as well as LSG hardware and suture removal as deemed appropriate, ensuring the stomach remains relatively distensible back to its normal configuration. Whether R-EG comes with this ability to allow for the conversion operation that would see the complete mobilization of the stomach fundus and the removal of any extra-gastric adhesions and any remaining visible LSG hardware to restore the anatomy of the stomach will be an area worth examining further in the future to inform the extent to which R-EG may be implemented and also give insight into any adverse events or additional safety concerns likely to accrue from the procedure. It is very important during this R-EG procedure to ensure that there is no more hardware in the trajectory of the staple line to avoid staple misfiring [19]. Another key observation from studies centering on R-EG's safety is that during stomach anatomy restoration, a rotated sleeve must be prevented to minimize the risk of proximal staple line leak or iatrogenic reflux [15].

Furthermore, practitioners should discern any stomach thickening relative to inflammatory pseudo polyps or muscular hypertrophy during R-EG and, if in doubt, opt for on-table endoscopy. To locate any undetected LSG hardware during R-EG, intra-operative endoscopic ultrasound has been recommended [5]. However, to justify the use of this procedure, there is a need for more data, especially relative to cost-effectiveness and the needed technical expertise.

## Conclusions

In summary, this review predicts that there might be an increasing trend in the implementation of R-EG in the future. The systematic review outcomes confirm the efficacy and safety of the procedure; however, during the investigation of any adverse events with which R-EG may be associated, a specific area worth studying comprehensively in the future include circumstances surrounding the restoration of the original stomach anatomy, as well as the need for scholarly studies to center on some of the meticulous techniques through which the majority or all of the LSG hardware could be identified and removed, hence ensuring successful and safe R-EG.
